# Transcranial focused ultrasound stimulation in schizophrenia: current evidence, mechanisms, and future directions

**DOI:** 10.3389/fpsyt.2026.1859036

**Published:** 2026-07-06

**Authors:** Chunyang Shi, Qing Zhao, Gang Zhang, Zhoubing Wang, Jun Zhou, Xianlu Chang, Ziming Liao, Wanying Zhang, Huihua Li, Huiying Xue, Kexu Zhang, Xiong Jiao, Junfeng Sun, Qiang Hu

**Affiliations:** 1 Department of Psychiatry, Zhenjiang Mental Health Center, Zhenjiang Fifth Affiliated Hospital of Jiangsu University, Zhenjiang, China; 2 Department of Psychiatry, Wuhu Hospital of Anding Hospital (The Fourth People’s Hospital of Wuhu), Wuhu, China; 3 School of Mental Health, Bengbu Medical University, Bengbu, China; 4 Department of Psychiatry, The Second Affiliated Hospital of Shenyang Medical College, Shenyang, China; 5 Department of Burn and Plastic Surgery, Zhenjiang Hospital of Chinese Traditional and Western Medicine, Zhenjiang, China; 6 Department of Psychiatry, Shandong Daizhuang Hospital, Jining, China; 7 Shanghai Key Laboratory of Mental Disorders Translational Research, Shanghai Mental Health Center, Shanghai Jiao Tong University School of Medicine, Shanghai, China; 8 Shanghai Med-X Engineering Research Center, School of Biomedical Engineering, Shanghai Jiao Tong University, Shanghai, China; 9 School of Mental Health and Psychological Sciences, Anhui Medical University, Hefei, China

**Keywords:** neuromodulation, non-invasive brain stimulation, schizophrenia, tFUS, transcranial focused ultrasound stimulation

## Abstract

**Background:**

Schizophrenia disrupts large-scale brain networks, especially circuits linking cortical and deep subcortical structures. Transcranial focused ultrasound stimulation (tFUS) is an emerging non-invasive neuromodulation technique with the capacity to target deep brain regions with high spatial precision. However, clinical evidence for tFUS in schizophrenia remains limited.

**Objective:**

This narrative review synthesizes available preclinical and clinical evidence, with particular attention to mechanisms, safety, stimulation targets, methodological limitations, and translational challenges.

**Current evidence:**

In dizocilpine (MK-801) rodent models, tFUS has been reported to reverse cognitive deficits, prevent psychotic-like behaviors, restore parvalbumin-positive GABAergic interneuron markers in the prefrontal cortex, and upregulate NMDA receptor-related signaling. In patients with schizophrenia, early studies suggest that tFUS is feasible and generally well tolerated. Reported targets include the dorsolateral prefrontal cortex (DLPFC) for negative symptoms, the globus pallidus/globus pallidus internus for sensory gating-related circuits, and striatal circuits for auditory hallucinations. Efficacy data remain preliminary.

**Challenges:**

Key challenges include bone-conducted auditory confounds that may compromise blinding, incomplete and heterogeneous stimulation-parameter reporting, small sample sizes, short follow-up periods, and limited evidence for durable clinical benefit.

**Future directions:**

Future studies should use rigorous sham or auditory-masking procedures, adopt standardized reporting frameworks such as the ITRUSST recommendations, and move from feasibility studies toward larger, circuit-specific, hypothesis-driven trials.

**Conclusion:**

Preclinical and early clinical studies suggest that tFUS may have therapeutic potential in schizophrenia, but current evidence is insufficient to support definitive clinical conclusions. Larger well-controlled studies are required.

## 1 Introduction

Schizophrenia affects approximately 1% of the population worldwide and is a chronic, disabling disorder (1). Patients may experience positive symptoms, negative symptoms, and cognitive impairment (2). Increasing evidence conceptualizes schizophrenia as a disorder of brain connectivity, characterized by dysfunction within large-scale neural networks (3). For example, hallucinations and delusions have been linked to impaired sensory gating, thalamocortical filtering abnormalities, and dysregulated subcortical dopamine signaling (4). Negative symptoms and cognitive impairment, by contrast, are commonly associated with reduced prefrontal-limbic engagement and altered synaptic connectivity (5). Although second-generation antipsychotics are effective for many patients, approximately one third respond inadequately and may meet criteria for treatment-resistant schizophrenia (6). One possible reason is that pharmacological treatment may not directly correct dysfunction in specific neural circuits (7). These unmet needs have encouraged the development of safer and more precise circuit-based interventions for schizophrenia.

Physical neuromodulation techniques are increasingly used in schizophrenia, but conventional non-invasive approaches have important constraints (8). Repetitive transcranial magnetic stimulation (rTMS) and transcranial direct current stimulation (tDCS) have shown therapeutic potential for selected symptom domains, but their effects are limited by penetration depth and spatial resolution (9, 10). These physical constraints make it difficult to engage deep nodes within cortico-striato-thalamo-cortical circuits, including the nucleus accumbens, globus pallidus, and thalamus (11). Other approaches, such as deep transcranial magnetic stimulation and temporal interference stimulation, have been developed to reach deeper structures. However, each modality has distinct advantages and limitations. Deep TMS can reach several centimeters beneath the scalp but has relatively broad spatial fields, whereas temporal interference stimulation offers steerable deep targeting through interfering electric fields that may be influenced by individual skull and scalp anatomy (12, 13). Transcranial focused ultrasound stimulation (tFUS) takes a different approach by using low-intensity mechanical waves that penetrate the skull and converge on target regions with high spatial precision (14, 15). This combination of depth and focality makes tFUS a promising, complementary approach for disorders involving deep circuit dysfunction, including schizophrenia.

Early clinical evidence supports the feasibility of this approach. A double-blind, randomized, sham-controlled study reported that 15 sessions of tFUS targeting the left dorsolateral prefrontal cortex (DLPFC) were associated with reductions in negative symptoms and improvements in cognitive performance in patients with chronic schizophrenia (16). More recent work has explored deeper subcortical targets, including globus pallidus circuits and striatal circuits implicated in auditory hallucinations (17, 18). A broader review has discussed the potential of tFUS across psychiatric disorders (12, 19). However, a focused synthesis of tFUS in schizophrenia remains needed because this disorder raises distinctive challenges, including deep target selection, symptom-circuit heterogeneity, auditory confounding, and incomplete parameter reporting (20).

## 2 Review methods

This narrative review synthesizes the available evidence on tFUS in schizophrenia. Given the limited number of available studies and substantial methodological heterogeneity, we used a structured literature search to improve transparency and reproducibility rather than conducting a formal systematic review or meta-analysis. PubMed, Web of Science, and PsycINFO were searched from database inception to February 2026. The search strategy combined terms related to ultrasound neuromodulation and schizophrenia: (“transcranial focused ultrasound” OR “tFUS” OR “focused ultrasound stimulation” OR “low-intensity focused ultrasound”) AND (“schizophrenia” OR “psychosis” OR “MK-801 model” OR “preclinical schizophrenia”). Reference lists of relevant articles and previous reviews were also screened manually.

Original research articles published in English or Chinese were eligible for inclusion. Both preclinical studies using rodent models of schizophrenia and clinical studies, including case series, feasibility studies, and controlled trials, were considered. Conference abstracts, editorials, opinion articles, and studies using high-intensity focused ultrasound for ablation rather than neuromodulation were excluded. From each included study, we extracted information on study design, sample size, target brain region, ultrasound parameters, main behavioral or clinical outcomes, neurophysiological or molecular findings, and reported adverse events. We did not perform a formal meta-analysis or structured risk-of-bias assessment because the evidence base was small and heterogeneous. Instead, methodological limitations, including sample size, sham adequacy, blinding integrity, follow-up duration, and translational relevance, are discussed throughout the review. We also specifically searched for studies reporting null, negative, or contradictory findings using the same databases and search strategy, but no eligible published studies meeting the inclusion criteria were identified. This may reflect publication bias in this emerging field, and we acknowledge this limitation.

## 3 Technical principles and system architecture of tFUS

### 3.1 Biophysical mechanisms

Low-intensity tFUS transmits ultrasonic waves through the skull. These waves typically use frequencies in the hundreds of kilohertz and converge on a defined brain target (14, 15). The neuromodulatory effects are thought to arise mainly from mechanical rather than thermal mechanisms (21, 22). Acoustic radiation forces and cyclic tissue displacement may exert stress on neuronal membranes and interact with mechanosensitive ion channels, including Piezo and transient receptor potential (TRP) channels (23–25). These effects may alter membrane polarization and neuronal excitability. Although some experimental work suggests that excitatory or inhibitory effects can depend on acoustic intensity, pulse duration, duty cycle, target region, and baseline neural state, the parameter-response relationship remains incompletely characterized (26, 27). Low-intensity protocols are designed to avoid ablative heating and cavitation, but thermal and mechanical safety should still be monitored carefully (26, 28).

This principle has already been translated into preclinical schizophrenia research. Animal models have shown that tFUS can modulate parvalbumin-positive interneuron-related markers in the prefrontal cortex (29, 30). These interneurons are implicated in excitation-inhibition imbalance in schizophrenia. Because tFUS effects depend on stimulation parameters and target circuits, future protocols may be tailored to symptom-specific mechanisms. For example, protocols that enhance prefrontal activity may be relevant to negative or cognitive symptoms, whereas protocols that reduce excessive striatal-temporal coupling may be relevant to hallucinations.

### 3.2 System composition of tFUS

A complete tFUS system has three main parts: an ultrasound generation unit, a neuronavigation system, and a monitoring module. Together they ensure precision, safety, and the ability to repeat the stimulation.

The ultrasound generation platform includes a control unit and a transducer. The control unit generates and adjusts ultrasound pulse parameters, including center frequency (typically 250 to 700 kHz), pulse repetition frequency (PRF, usually 10 to 1000 Hz), pulse width (0.1 to 10 ms), duty cycle (1 to 50%), and spatial peak temporal average intensity (0.1 to 30 W/cm²) (14). The transducer turns electrical signals into mechanical vibrations that produce ultrasound waves. There are two types based on how they focus. Single element concave transducers have a fixed focal length. Phased array transducers use electronic focusing and can adjust the focal point on the fly (31). A phased array system contains hundreds of independent elements. By changing the phase delay of each element, researchers can flexibly shape and move the focal spot. This ability is crucial for avoiding areas of the skull that vary in thickness or density and for getting energy to the target efficiently (32).

The monitoring and safety module has three functions. First is intracranial acoustic field monitoring. Acoustic simulations use individual CT or MRI data to model how the skull transmits sound and how the beam focuses. This predicts the focal position and intensity distribution (33, 34). Second is thermal monitoring. Infrared cameras or MRI thermometry sequences can be used to check scalp or tissue temperature and ensure that exposure remains within safe limits (35). Third is neurophysiological monitoring. Synchronized recordings of electroencephalography (EEG), functional near-infrared spectroscopy (fNIRS), or functional MRI (fMRI) allow real-time assessment of neural responses and provide feedback for stimulation-parameter optimization (36, 37).

## 4 Existing research on tFUS treatment for schizophrenia

The evidence base for tFUS in schizophrenia currently includes two preclinical studies and three clinical studies. These studies differ substantially in design, target selection, stimulation parameters, and outcome measures. To help readers navigate this heterogeneous literature, we summarize the key characteristics of each study in **Table 1**.

**Table 1:** Summary of included studies on tFUS in schizophrenia.

Study	Design/sample	Target	Protocol	Main findings, safety, and limitations
Tsai et al., 2022 (29)	Preclinical; MK-801 rats; sample NR in this review	Prefrontal/cingulate circuits	Single session; frequency/complete acoustic parameters NR	Improved cognitive inflexibility; restored PV+/calbindin and BDNF markers; no AEs reported; limited long-term safety data.
Pan et al., 2024 (30)	Preclinical; MK-801 rats; sample NR in this review	Prefrontal cortex	500 kHz; 176 kPa peak pressure; 5 min before MK-801	Prevented hyperlocomotion, social withdrawal, and cognitive deficits; NR1/BDNF upregulated; no AEs reported; preventive acute model.
Zhai et al., 2023 (16)	Double-blind randomized sham-controlled study; 32 randomized/26 analyzed	Left DLPFC	500 kHz; 10% duty cycle; 15 sessions over 3 weeks	Reduced SANS negative symptoms and reported cognitive improvement; mild headache; limited effect-size and blinding details.
Brinker et al., 2025 (17)	Feasibility/safety report in dynamic clinical settings	Globus pallidus/GPi circuit	Dynamic clinical tFUS strategy; parameters limited in brief report	Feasible deep targeting; no serious AEs; primarily safety/feasibility focused, with efficacy preliminary.
Subramaniam et al., 2026 (18)	First-in-human feasibility study; 3 patients	Striatal circuits including nucleus accumbens and caudate head	I_sppa_:7.72-22.86 W/cm²; MI < 1.9; single session	Reduced hallucination severity and striatal-temporal connectivity; safe; unfocused sham helped preserve blinding; exploratory.

### 4.1 Preclinical evidence

In 2022, Tsai and colleagues used a dizocilpine (MK-801) rodent model of schizophrenia and applied tFUS after behavioral abnormalities had emerged (29). The tFUS improved cognitive inflexibility, particularly reversal-learning deficits. Several neurobiological changes accompanied these behavioral improvements. The animals showed restored expression of parvalbumin (PV) in the prefrontal/cingulate cortical region. PV-positive interneurons are a subtype of GABAergic neurons that help maintain excitation-inhibition balance in cortical circuits, and their dysfunction is strongly implicated in schizophrenia. The study also reported changes in calbindin and brain-derived neurotrophic factor (BDNF), suggesting that tFUS may influence GABAergic interneuron-related pathology. These findings provide preliminary proof-of-concept evidence, although the study used a single pharmacological model and did not provide long-term safety data.

The same research group later examined whether tFUS could prevent MK-801-induced abnormalities when applied before pharmacological challenge (30). This preventive approach attenuated hyperlocomotion, social withdrawal, and cognitive deficits. At the molecular level, the protective effects were accompanied by upregulation of the NMDA receptor subunit NR1 and BDNF in the prefrontal cortex. These findings extend the preclinical rationale from symptom reversal to prevention, but their translational relevance remains limited by reliance on acute MK-801 models rather than chronic or neurodevelopmental models of schizophrenia.

These preclinical findings offer several lessons for clinical study design. First, the restoration of PV-positive interneuron-related markers supports the prefrontal cortex as a plausible target for symptoms involving cortical excitation-inhibition imbalance. Second, the possibility of tuning tFUS effects through acoustic parameters may enable symptom-specific protocols. Third, preventive effects in animals raise the hypothesis that tFUS might eventually be tested in clinical high-risk populations, although such use remains speculative. Future preclinical work should evaluate more chronic and neurodevelopmental models, define parameter-response relationships, and report acoustic parameters in a standardized manner.

### 4.2 Clinical research

Clinical applications of tFUS in schizophrenia remain at an early stage. The available studies support technical feasibility and short-term tolerability, but they do not yet establish durable clinical efficacy.

Zhai and colleagues conducted a double-blind, randomized, sham-controlled study targeting the left DLPFC in patients with chronic schizophrenia (16). Thirty-two participants were randomized and 26 were included in the final analysis. The active group received 15 sessions over three weeks. Compared with the sham group, active tFUS was associated with reductions in negative symptoms measured by the Scale for the Assessment of Negative Symptoms (SANS), as well as cognitive improvement. This study provides encouraging preliminary evidence, but its brief-report format limits details on effect sizes, blinding integrity, and durability of response.

A subsequent feasibility and safety report from Brinker and colleagues described tFUS in dynamic clinical settings, including a strategy for targeting the globus pallidus/globus pallidus internus circuit in schizophrenia (17). This work is important because conventional cortical stimulation cannot directly reach such deep basal ganglia structures. However, the study primarily supports technical feasibility and short-term safety rather than clinical efficacy. The therapeutic relevance of GPi or globus pallidus targeting for psychotic symptoms requires confirmation in adequately powered, sham-controlled trials.

Most recently, Subramaniam and colleagues reported a first-in-human feasibility study targeting striatal circuits in three patients with schizophrenia and auditory hallucinations (18). The study used an unfocused ultrasound sham condition to help preserve blinding. Active stimulation was safe in this small sample and was associated with reductions in hallucination severity and altered striatal-temporal functional connectivity. Because the trial was exploratory and included only three patients, these findings should be interpreted as proof-of-mechanism and hypothesis-generating rather than definitive evidence of efficacy.

Taken together, these pioneering studies suggest that tFUS can be delivered to both cortical and deep subcortical targets relevant to schizophrenia. Reported targets include the DLPFC for negative symptoms, globus pallidus circuits for sensory gating-related pathways, and striatal circuits for hallucinations. At present, however, the clinical evidence mainly supports feasibility and short-term tolerability. Larger controlled trials with rigorous sham procedures, standardized parameter reporting, and longer follow-up are needed before treatment efficacy can be established.

## 5 Safety and tolerability of tFUS

Available clinical studies suggest that low-intensity tFUS is generally well tolerated when delivered within appropriate safety parameters. Systematic reviews of human and animal studies report that adverse effects are usually mild and transient, although the evidence base remains relatively small and heterogeneous (38, 39).

The most commonly reported adverse effect is mild headache (39, 40). Other reported effects include transient scalp warmth, fatigue, nausea, dizziness, tingling, neck discomfort, or anxiety (39–41). Serious adverse events such as intracranial hemorrhage, seizures, or persistent neurological deficits have not been commonly reported in low-intensity protocols, but rare risks cannot be excluded from the available sample sizes (38–41). Safety assessment should therefore include systematic adverse-event monitoring, acoustic exposure reporting, and longer-term follow-up, particularly in patient populations receiving repeated sessions.

The limited schizophrenia-specific evidence is consistent with short-term tolerability. Zhai and colleagues reported that repeated DLPFC stimulation was well tolerated in patients with chronic schizophrenia (16). Brinker and colleagues reported no serious adverse events in dynamic clinical settings involving deep targets (17). Subramaniam and colleagues reported detailed acoustic parameters, including I_SPPA_ values of 7.72-22.86 W/cm² and MI < 1.9, and used an unfocused ultrasound sham protocol when targeting striatal circuits (18). Nevertheless, long-term safety after repeated or multi-target stimulation in schizophrenia remains insufficiently characterized.

## 6 Discussion

Schizophrenia is a chronic and often treatment-refractory disorder for which current pharmacological and physical interventions remain insufficient for many patients (42, 43). The rTMS and tDCS have shown benefits for selected symptoms, but their clinical application is constrained by limited depth and spatial precision (44, 45). The tFUS may help address these limitations by enabling non-invasive, focal modulation of cortical and subcortical circuits using low-intensity acoustic energy (14, 46, 47). Rather than replacing existing neuromodulation approaches, tFUS should be viewed as a complementary strategy with a distinct physical mechanism and target profile.

### 6.1 Summary of emerging evidence

In animal models, the evidence has moved from reversal of MK-801-induced abnormalities to prevention of their emergence. The 2022 study reported that tFUS improved cognitive inflexibility and modulated calcium-binding proteins and BDNF-related markers (29). The 2024 study applied tFUS before MK-801 exposure and reported attenuation of locomotor, social, and cognitive abnormalities, accompanied by changes in NMDA receptor-related signaling and BDNF (30). Together, these studies provide a coherent preclinical rationale for tFUS, but they remain limited by the use of acute pharmacological models.

On the clinical side, available studies remain sparse. The DLPFC study suggests potential benefit for negative symptoms (16), whereas the globus pallidus and striatal studies highlight the feasibility of engaging deeper circuits (17, 18). These studies support the technical promise of tFUS in schizophrenia, but clinical efficacy remains uncertain because sample sizes are small, follow-up is short, stimulation targets differ, and sham/blinding procedures are incompletely standardized.

Several major limitations remain. First, the current clinical evidence is not powered to establish durable efficacy. Second, the studies used different targets and protocols, making cross-study comparison difficult. Third, the biological pathway linking acoustic stimulation, circuit modulation, and symptom improvement remains incompletely defined. These limitations justify further development of tFUS in schizophrenia, but they also argue against premature clinical claims.

### 6.2 Toward optimized stimulation: targeting symptom-specific circuits

Schizophrenia is heterogeneous, and future tFUS protocols will likely need to align stimulation targets with symptom-specific circuits. However, the optimal target for each symptom domain remains unresolved.

Different symptoms of schizophrenia may map onto partially distinct brain circuits. Positive symptoms such as hallucinations and delusions have been linked to abnormalities in thalamocortical, temporoparietal, and striatal circuitry. Subramaniam and colleagues reported that striatal-targeted tFUS was associated with reduced hallucination severity and altered striatal-temporal connectivity (18). Negative symptoms may involve prefrontal, anterior cingulate, and ventral striatal circuits, whereas cognitive impairment may involve prefrontal-hippocampal dysfunction (48–52). These circuit hypotheses require direct testing in adequately powered target-comparison studies.

Future trials should test symptom-circuit links directly. For example, studies could compare cortical DLPFC stimulation with anterior cingulate or striatal targets for negative symptoms, or compare globus pallidus and striatal targets for hallucinations and sensory-gating abnormalities. Such designs would move the field from feasibility studies toward personalized, symptom-guided tFUS.

### 6.3 Standardization and confound control

A major challenge in tFUS research is the lack of standardized methods. Frequencies, intensities, duty cycles, pulse durations, and sonication schedules vary widely across studies. The ITRUSST consensus on standardized reporting recommends detailed reporting of the transducer and drive system, drive settings, free-field acoustic parameters, pulse timing parameters, *in situ* exposure estimates, and intensity parameters (53). Future schizophrenia trials should report key variables such as spatial peak pulse average intensity (I_SPPA_), spatial peak temporal average intensity (I_SPTA_), mechanical index (MI), *in situ* derated pressure, duty cycle, sonication duration, targeting method, and sham procedure. Without this level of reporting, reproducible dose-response relationships cannot be established.

Auditory confounding is equally important. tFUS can generate bone-conducted sound that activates auditory pathways and arousal networks (20, 54, 55). This sensory cue can compromise blinding and may produce non-specific effects. The issue is particularly relevant in schizophrenia because sensory gating is often impaired. Future trials should therefore incorporate active auditory masking, acoustic-matched controls, or unfocused ultrasound sham procedures where appropriate. Subramaniam and colleagues used an unfocused sonication sham that created a diffuse pressure field without focused energy delivery, representing an important methodological advance for preserving blinding (18).

A third challenge is personalized dosimetry (53, 56). Skull thickness, density, and porosity can substantially affect ultrasound transmission and focal energy deposition. Even with identical device settings, the actual intracranial exposure may vary between individuals. Patient-specific CT- or MRI-based acoustic modeling, phased-array focusing, and real-time monitoring may help reduce this variability, but these procedures increase complexity and are not yet standardized for routine psychiatric use.

### 6.4 Comparison with other deep-targeting neuromodulation approaches

Other neuromodulation techniques have also been developed to engage deeper brain structures. Deep transcranial magnetic stimulation can reach several centimeters beneath the scalp but provides relatively limited focal precision because of its broad stimulation field (12). Temporal interference stimulation uses interfering electric fields from surface electrodes to steer modulation toward deeper targets, but the resulting field distribution may be influenced by individual skull and scalp anatomy (13). Compared with these approaches, tFUS offers mechanical neuromodulation with high spatial precision and the ability to focus acoustic energy at cortical or subcortical targets (14, 15, 46).

These distinctions should not be interpreted as evidence that tFUS is categorically superior. The tFUS has its own limitations, including the need for individualized acoustic modeling, auditory confounding, and the lack of standardized parameter settings (20, 53–56). Rather than claiming superiority, tFUS may complement current neuromodulation approaches in situations where precise, focal, and non-invasive modulation of deep neural circuits is required.

### 6.5 Methodological limitations and publication bias

Several methodological limitations should be considered when interpreting the current evidence. First, the available studies are characterized by uniformly small sample sizes. The clinical studies ranged from 3 to 32 participants, limiting statistical power and the precision of effect estimates (16–18). Second, most studies were exploratory in design and were powered primarily for feasibility or safety rather than definitive clinical efficacy (17, 18). Third, sham adequacy and blinding integrity were not consistently reported across studies. Because tFUS inevitably produces bone-conducted sound, inadequate control of these sensory cues may compromise blinding and potentially influence participant expectations and study outcomes (20, 54, 55). Additional limitations include short follow-up periods and limited translational validity. No clinical study has reported long-term outcomes beyond one month, leaving the durability of treatment effects uncertain. Furthermore, current preclinical evidence relies heavily on acute MK-801 models, which capture only selected features of schizophrenia and may not fully reflect its neurodevelopmental nature (29, 30, 57, 58). These limitations indicate that the current findings should be considered preliminary and hypothesis-generating.

Potential publication bias should be considered when interpreting the current evidence. Most available studies report positive or supportive findings, whereas no eligible published studies with clearly negative results were identified despite targeted searches. This absence should not be interpreted as evidence of consistent efficacy, because unpublished negative studies, selective outcome reporting, and small-sample publication bias remain possible. Therefore, the current evidence base should be interpreted cautiously until larger, well-controlled studies become available.

### 6.6 Practical barriers to clinical translation

Even if tFUS proves efficacious in larger trials, several practical barriers stand in the way of routine clinical use. Compared with established non-invasive neuromodulation techniques such as rTMS and tDCS, current tFUS systems require more complex infrastructure, including phased-array transducers, neuronavigation platforms, and access to structural imaging for individualized acoustic modeling (53, 56). These requirements increase both the financial cost and logistical complexity of treatment delivery. Operating tFUS also requires specialized expertise in ultrasound physics, transducer positioning, acoustic stimulation, acoustic modeling, and real-time monitoring. Interindividual variability in skull density, thickness, and porosity can substantially influence ultrasound transmission and focal energy deposition, making patient-specific acoustic modeling important for safety and reproducibility.

Reproducibility is another barrier. Across the studies summarized in **Table 1**, stimulation targets and parameters vary widely. Without consensus on optimal settings, different centers may obtain different results, slowing clinical adoption. Patient throughput may also be limited because each session requires setup, targeting, and monitoring. These barriers suggest that early tFUS adoption will likely be limited to specialized academic or research centers with multidisciplinary engineering, imaging, and clinical support. Reducing cost, automating acoustic modeling, and developing simplified protocols are priorities for broader dissemination (59).

### 6.7 Future directions

Despite these practical barriers, several promising directions could accelerate the translation of tFUS into clinical practice. Research on tFUS in schizophrenia remains at an early stage. To establish therapeutic efficacy beyond technical feasibility, future studies should move toward circuit-based precision psychiatry.

One priority is closed-loop neuromodulation. Schizophrenia is characterized by dynamic abnormalities in connectivity within deep brain circuits. Conventional open-loop stimulation may not fully account for ongoing changes in neural activity. Combining tFUS with synchronous neuroimaging or electrophysiological monitoring could enable adaptive stimulation, in which ultrasound pulses are delivered when abnormal circuit states are detected (60, 61). Another direction is early intervention. Preclinical findings suggest that tFUS may prevent MK-801-induced behavioral abnormalities, but whether this concept can be translated to clinical high-risk populations remains unknown (30).

As the technology improves, tFUS may become an additional investigational option for patients with treatment-resistant schizophrenia. Its ability to modulate deep circuits non-invasively makes it a promising approach for further study, but it should be evaluated through larger sham-controlled trials with standardized reporting, rigorous blinding assessment, and long-term safety follow-up.
